# Porous Coatings Containing Copper and Phosphorus Obtained by Plasma Electrolytic Oxidation of Titanium

**DOI:** 10.3390/ma13040828

**Published:** 2020-02-12

**Authors:** Krzysztof Rokosz, Tadeusz Hryniewicz, Wojciech Kacalak, Katarzyna Tandecka, Steinar Raaen, Sofia Gaiaschi, Patrick Chapon, Winfried Malorny, Dalibor Matýsek, Kornel Pietrzak, Ewa Czerwińska, Anna Iwanek, Łukasz Dudek

**Affiliations:** 1 Faculty of Mechanical Engineering, Koszalin University of Technology, Racławicka 15-17, PL 75-620 Koszalin, PolandKatarzyna.Tandecka@tu.koszalin.pl (K.T.); kornel.pietrzak@s.tu.koszalin.pl (K.P.);; 2 Department of Physics, Norwegian University of Science and Technology (NTNU), Realfagbygget E3-124 Høgskoleringen 5, NO 7491 Trondheim, Norway; steinar.raaen@ntnu.no; 3 HORIBA FRANCE S.A.S., Avenue de la Vauve - Passage Jobin Yvon, CS 45002-91120 Palaiseau, France; sofia.gaiaschi@horiba.com (S.G.); patrick.chapon@horiba.com (P.C.); 4 Hochschule Wismar-University of Applied Sciences Technology, Business and Design, Faculty of Engineering, DE 23966 Wismar, Germany; winfried.malorny@hs-wismar.de; 5 Institute of Geological Engineering, Faculty of Mining and Geology, VŠB—Technical University of Ostrava, 708 33 Ostrava, Czech Republic; dalibor.matysek@vsb.cz

**Keywords:** plasma electrolytic oxidation (PEO), micro arc oxidation (MAO), titanium, copper(II) nitrate(V) trihydrate, orthophosphoric acid, antibacterial and antifungal coatings

## Abstract

To fabricate porous copper coatings on titanium, we used the process of plasma electrolytic oxidation (PEO) with voltage control. For all experiments, the three-phase step-up transformer with six-diode Graetz bridge was used. The voltage and the amount of salt used in the electrolyte were determined so as to obtain porous coatings. Within the framework of this study, the PEO process was carried out at a voltage of 450 V_RMS_ in four electrolytes containing the salt as copper(II) nitrate(V) trihydrate. Moreover, we showed that the content of salt in the electrolyte needed to obtain a porous PEO coating was in the range 300–600 g/dm^3^. After exceeding this amount of salts in the electrolyte, some inclusions on the sample surface were observed. It is worth noting that this limitation of the amount of salts in the electrolyte was not connected with the maximum solubility of copper(II) nitrate(V) trihydrate in the concentrated (85%) orthophosphoric acid. To characterize the obtained coatings, numerous techniques were used. In this work, we used scanning electron microscopy (SEM) coupled with electron-dispersive X-ray spectroscopy (EDS), conducted surface analysis using confocal laser scanning microscopy (CLSM), and studied the surface layer chemical composition of the obtained coatings by X-ray photoelectron spectroscopy (XPS), X-ray diffraction (XRD), glow discharge of optical emission spectroscopy (GDOES), and biological tests. It was found that the higher the concentration of Cu(NO_3_)_2_∙3H_2_O in the electrolyte, the higher the roughness of the coatings, which may be described by 3D roughness parameters, such as *Sa* (1.17–1.90 μm) and *Sp* (7.62–13.91 μm). The thicknesses of PEO coatings obtained in the electrolyte with 300–600 g/dm^3^ Cu(NO_3_) _2_∙3H_2_O were in the range 7.8 to 10 μm. The Cu/P ratio of the whole volume of coating measured by EDS was in the range 0.05–0.12, while the range for the top layer (measured using XPS) was 0.17–0.24. The atomic concentration of copper (0.54–0.72 at%) resulted in antibacterial and fungicidal properties in the fabricated coatings, which can be dedicated to biocompatible applications.

## 1. Introduction

In recent years, apart from the development of the plasma electrolytic oxidation (PEO) process, many other techniques allowing surface functioning for biomedical purposes have been elaborated. Fabrications of porous coatings on titanium or its alloys by means of the plasma electrolytic oxidation (PEO) method are mostly performed in aqueous electrolytes, with the addition of different salts that results in the formation mainly of titanium (IV) oxides (anatase and/or rutile) enriched with elements from the electrolyte. It should be noted that additives of salts, acids, or alkalis in amounts of a few up to several dozen grams per liter, as well as other parameters of the processes (electrode sparing voltage, stabilization of voltage or current, frequency of voltage or current) affect both the changes in chemical or phase composition of the obtained coating and their mechanical, electrochemical, and biological properties, which are important in designing machine parts, biocompatible materials, and catalysts as well as coatings resistant to attrition and wear. Numerous studies show that coatings obtained by plasma electrolytic oxidation (PEO) may undergo functionalization through additional treatments, improving their biocompatible [1,2,3,4], bactericidal properties. The aim of this paper is to present novel phosphorus coatings obtained on titanium and enriched with copper for biomedical use.

Coatings enriched with copper, coming from copper compounds or nanoparticles located in the electrolyte, exhibit antibacterial properties. Such coatings may be obtained, among other methods, by a four-minute PEO process (16.5 A/dm^2^, 800 Hz, 10%) in the electrolyte based on an aqueous solution containing Ca(CH_3_COO)_2_·H_2_O, C_3_H_7_Na_2_O_6_P·5H_2_O, and Cu(CH_3_COO)_2_. The resulting layers, containing TiO_2_ (anatase, rutile) and Cu^2+^ ions, were characterized by a porous structure of craters with diameters of 3 to 5 µm, and the presence of copper in the coating had no significant effect on the change of surface topography and the phase composition of coatings. Moreover, studies have shown that the coatings are characterized by antibacterial properties and are non-toxic for living organisms and, in addition, adhesion and osteoblastic growth occur here faster than on coatings that do not contain copper(II) ions [5].

When using voltage stabilization during the PEO process (450 V, 100 Hz, 26%) in the same electrolyte, porous coatings enriched with copper (0.67–1.93 wt%) were also obtained, containing crystal phases of anatase and rutile as well as an amorphous phase probably enriched in Ca_3_(PO_4_)_2_ and/or CaHPO_4_ and Cu^2+^ ions. It was shown that the addition of copper(II) acetate did not affect the morphology of obtained surfaces, but did have an effect on the antibacterial properties of fabricated layers. Moreover, it was noted that the amount of copper equal to 1.93 wt% in the coating, in addition to its antibacterial properties, is characteristic of cytotoxicity [6].

In the five-minute PEO process (20 A/dm^2^) in aqueous electrolyte containing NaH_2_PO_4_, NaOH, and copper nanoparticles, the antibacterial porous coatings were fabricated, containing both rutile and anatase forms in their structure. It was demonstrated that copper nanoparticles are both on the surface of the coating and inside its pores, such that the resulting coatings were characterized by good antimicrobial properties [7].

Our previous research studies were the first to take advantage of electrolytes based on concentrated orthophosphoric acid to fabricate coatings in the PEO processing [8,9,10]. The use of Cu(NO_3_)_2_∙3H_2_O salt with concentrated H_3_PO_4_ (85 wt%) as the electrolyte in the PEO process allowed us to obtain the extended surface structures of the porous characteristics containing compounds of copper and phosphorus on the Ti6Al4V alloy surface. The researchers indicated that the content of copper(II) nitrate(V) trihydrate essentially affected both the surface geometry and the composition of the obtained coatings [11,12,13,14,15,16].

The goal of this work is to fabricate porous antibacterial and fungicidal coatings, which could be used in biomedical applications. In this way, they should be as similar as possible to hydroxyapatite, with the addition of copper ions in their structure. On the other hand, the content of copper should be limited to a minimum in order to maintain good antibacterial properties.

## 2. Materials and Methods

For this work, 10 × 10 × 2 mm CP Titanium Grade 2 sheets (delivered by Bimo Tech Co., Wrocław, Poland) were used. In this paper, abbreviated sample names will be used as is shown in Table 1. The set-up consisted of a high-power DC voltage source (home-made three-phase power with a Graetz bridge of 450 V_RMS_) and a reaction cell with the home-made cylindrical cathode (cylinder of AISI 316L SS; diameter of 90 mm). Images of the coatings were obtained by means of a FEI Quanta 250 scanning electron microscope (Field Electron and Iron Company, Hillsboro, OR, USA). Analysis of the atomic composition of the obtained coatings was carried out by means of energy-dispersive X-ray spectroscopy (EDS Silicon Drift Detectors), using the ThermoScientific Co. ultra-dry EDS detector (Keith Thompson, Thermo Fisher Scientific, Madison, WI, USA). The XPS studies were performed by means of the SCIENCE SES 2002 apparatus (SCIENTA AB, ScientaOmicron, Uppsala, Sweden), taking advantage of a monochromatic source of X-ray radiation Al Kα (*hν* = 1486.6 eV). All the presented XPS spectra have been calibrated against the binding energies of C–C and C–H for carbon (C1s), equaling 284.8 eV, and the analyses of the obtained data were carried out using Casa XPS 2.3.14 (Casa Software Ltd., Teignmouth, Devon, UK) and MATLAB 2017a software (MathWorks, Inc., Natick, MA, USA). To analyze the crystal phase, XRD studies were carried out using the Bruker’s Co. D8 Advance measuring system (BRUKER Corporation, Billerica, MA, USA), equipped with the X-ray radiation source Cu Kα (40 kV, 40 mA). The surface topography was performed by using a LEXT OLS 4000 confocal laser microscope (Olympus Co., Japan). The total dimension of the surface profiles under geometrical analysis used to determine the selected 3D parameters for the evaluation of surface roughness was equal to 256 × 256 µm (0.0656 cm^2^). To describe the surface, we used altitude parameters such as the height of the highest surface elevation, *Sp*, and the arithmetic mean/mean average of the surface height, *Sa*, in accordance with the ISO 25178 standard. The elemental depth profile was obtained by glow discharge optical emission spectroscopy (GDOES, HORIBA Scientific, Palaiseau, France). For this purpose, a Horiba Scientific GD Profiler 2 (HORIBA Scientific, Palaiseau, France) was used, equipped with a 4 mm diameter anode.

The antimicrobial effects of coatings were assayed using a direct method, based on the criteria contained in the description of the SN 195920-ASTM E2922 standard. A grafted suspension of the microorganisms at a concentration of 10^8^ cfu/mL (colony forming unit per milliliter; 0.5 McFarland standard as determined by a densitometer DN 1B MF, BIOSAN, Riga, Latvia) was placed on the surface of the medium. After 20 min, the biomaterials of the coatings being tested in this study were directly incubated with substrate at 37 °C for 24 h. The study used two species of microbes derived from the American Type Culture Collection: *Candida albicans* NCPF 3255/ATCC 2091, and *Escherichia coli* NCTC 12241/ATCC 25922. After incubation, growth inhibition zones of the microorganisms tested were assessed. The presence of a growth-inhibiting zone around the tested microorganisms was the assessment criterion for bactericidal and fungicidal properties.

For cell adhesion tests after breeding, the discs were washed three times with sterile distilled water to remove non-adhesion cells, and were then shaken for a period of 15 min in distilled water. After drying the coatings, bis benzidine dye was applied to the surface (to observe living cells—intense yellow color) at 0.5 cm^3^/120 s. After rinsing in distilled water, propyl iodine was applied (observation of dead cells—orange color), 0.3 cm^3^ for 120 s, and again washed with water. Air-dried samples were observed in a fluorescent microscope (Motic BA 410 E M MOTIC, Hong Kong, Asia). The total viable cell count average of ten fields of view with a surface area of 1 mm^2^ was calculated by using the Motic Live Imaging module (Motic China Group CO, Motic Asia Hong Kong).

## 3. Results

**Figure 1:**
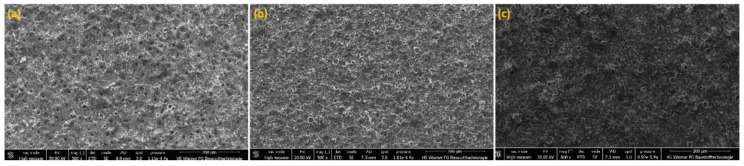
SEM images of plasma electrolytic oxidation (PEO) coatings of the three coatings TiCu300 (**a**), TiCu450 (**b**), TiCu600 (**c**).

**Figure 2:**
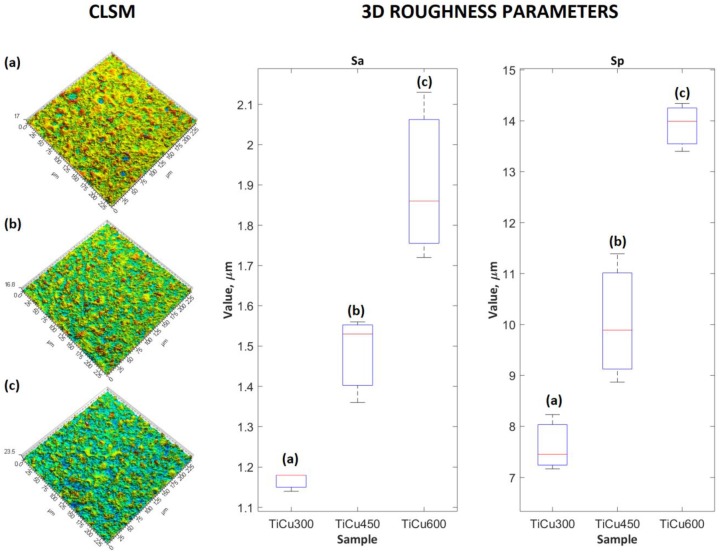
CLSM images of PEO coatings (left side) and corresponding to 3D surface roughness parameters (right side) of the three coatings: TiCu300 (**a**), TiCu450 (**b**), and TiCu600 (**c**).

Three samples of PEO coatings were analyzed, formed on titanium in electrolytes containing from 300 to 600 g/dm^3^ of Cu(NO_3_)_2_∙3H_2_O, due to their chemical and phase composition as well as their surface roughness. The SEM and CLSM images of the coating surfaces after PEO processes in electrolytes with different content of copper(II) nitrate(V) are presented in Figure 1 and Figure 2. It was found that with increases of salt in the electrolyte, the sharpness of pores also increased, resulting in greater development of the surface. Quantitative analysis using 3D parameters (*Sa* and *Sp*) to describe the surface roughness clearly indicates that increasing the amount of salt (Cu(NO_3_)_2_∙3H_2_O) from 300 to 600 g/dm^3^ in the solution resulted in an increase in both *Sa* and *Sp* from 1.17 ± 0.02 μm to 1.90 ± 0.21 μm and from 7.62 ± 0.55 μm to 13.91 ± 0.48 μm, respectively, as displayed in Figure 2.

**Figure 3:**
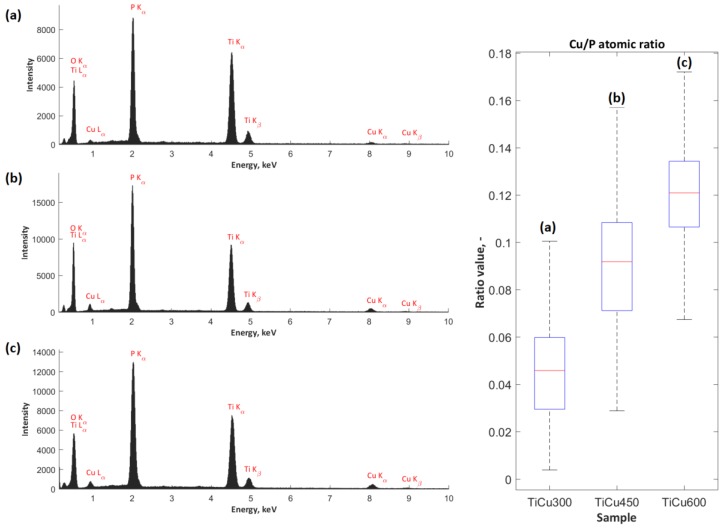
The EDS spectra of PEO coatings (left side) and corresponding copper-to-phosphorus (Cu/P) ratios (right side) of the three coatings: TiCu300 (**a**), TiCu450 (**b**), and TiCu600 (**c**).

The exemplary EDS spectra, obtained from the analysis of the surface chemical composition of coatings (TiCu300, TiCu450, TiCu600) under magnification of 500×, are presented in Figure 3. For each set of spectra for the porous coatings, peaks corresponding to the presence of copper (Cu L_α_, Cu K_α_, and Cu K_β_), phosphate (P K_α_), and titanium (Ti K_α_, Ti K_β_) were detected. The characterization of the amount of copper in the studied coatings allowed for quantitative analysis. For this purpose, the atomic ratio of the percentage content of copper-to-phosphorus (Cu/P) was determined, revealing the growth from 0.05 ± 0.02 to 0.12 ± 0.02. The higher content of copper in the coating volume, revealed by the EDS technique, allows us to assume that growth of copper content also occurred in the top layer of the obtained coating. However, the EDS method does not provide complete quantitative and qualitative information related to the studied coatings; this can be completed with XPS measurements.

**Figure 4:**
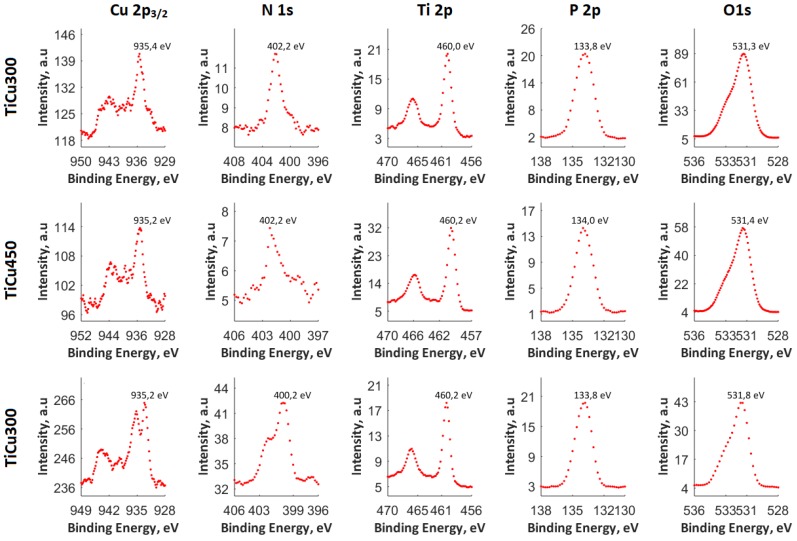
The XPS spectra of the three coatings: TiCu300, TiCu450, and TiCu600.

**Figure 5:**
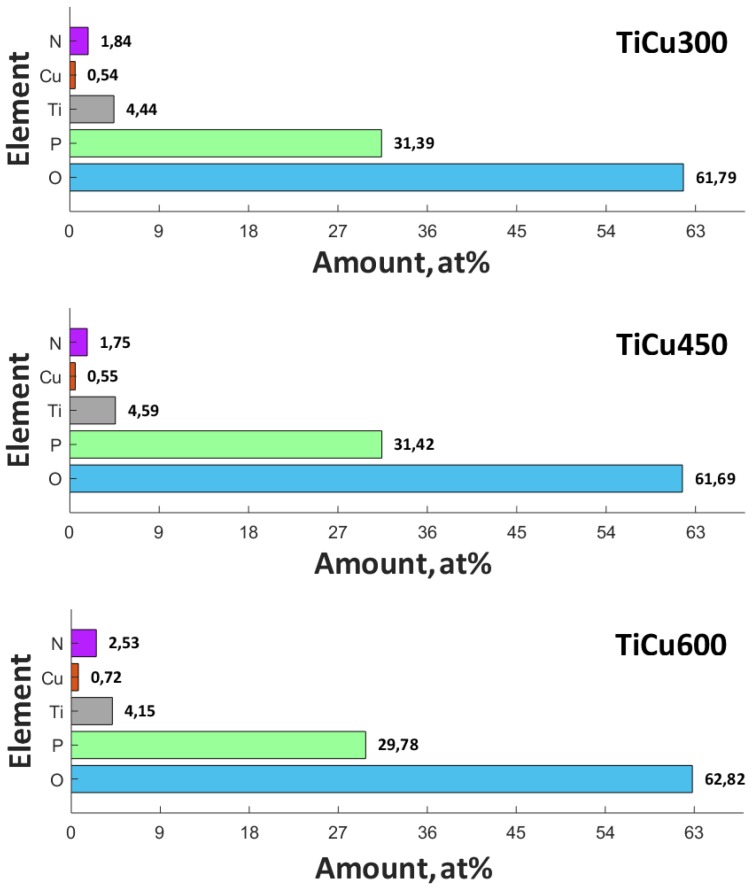
The quantitative analyses of XPS results of the three coatings: TiCu300, TiCu450, and TiCu600.

For this purpose, XPS studies were carried out with the results with spectra displayed in Figure 4. As opposed to the results of the XPS analyses carried out on the coatings fabricated in electrolytes containing the addition of Cu(NO_3_)_2_∙3H_2_O amounting to 300 g/dm^3^, for which the ratio Cu/Ti equaled 0.122, in the case of the surface layer of the PEO coating, obtained in the electrolyte containing the copper(II) nitrate(V) trihydrate of 600 g/dm^3^ concentration, the ratio of Cu/Ti increased to 0.174. The binding energies, equaling 460.0–460.2 eV (Ti 2p_3/2_) and 935.5–935.4 eV (Cu 2p_3/2_), suggest the presence of mainly Ti^4+^, Cu^+^, and/or Cu^2+^ in the top layer. The position of the peaks of phosphorus P 2p (133.8–140.0 eV) and oxygen O 1s (531.3–531.8 eV) indicates the possibility of phosphate or/and diphosphate occurrence. The spectra of nitrogen N 1s (402.2 eV) should be interpreted, rather, as contamination of organic derivation: both atmospheric and having been introduced during sample preparation for the analysis. The maximum position of N 1s (402.2 eV) does not exclude the possibility of the inception of ammonium compounds of non-organic origin into the surface layer of the obtained coating. It was recorded that in the top layer of PEO coatings, copper, titanium, nitrogen, phosphorus, and oxygen are in the range of 0.54-0.72, 4.15–4.44, 1.75–2.53, 29.78–31.42, and 61.69–62,82, respectively, as displayed in Figure 5. In addition, the proportion of the content Ti/P in the obtained surface layer belongs to the range 0.141 to 0.146, whereas Cu/P belongs to the range 0.017 to 0.24.

**Figure 6:**
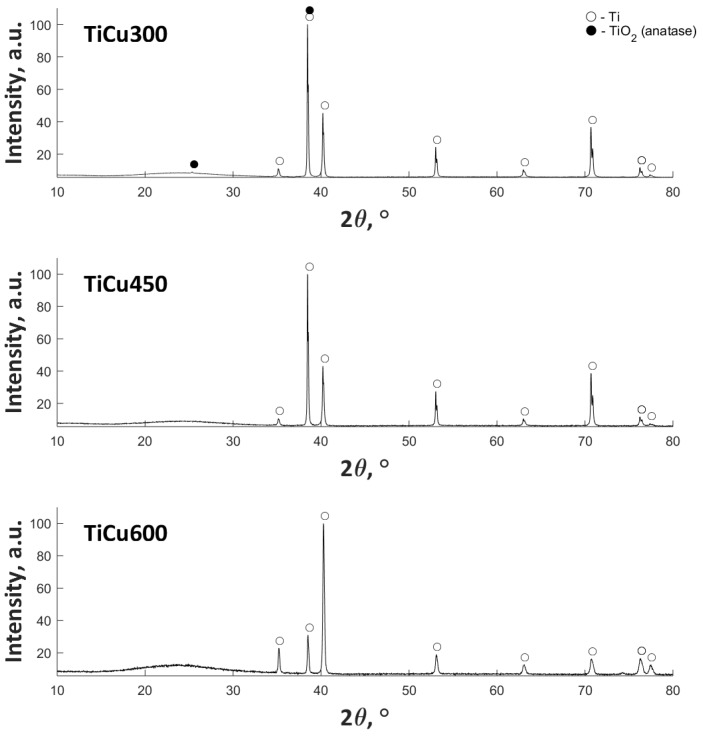
XRD diffractograms of PEO coatings (left side) and corresponding copper-to-phosphorus (Cu/P) ratios (right side) of the three coatings: TiCu300 (**a**), TiCu450 (**b**), and TiCu600 (**c**).

The XRD results (Figure 6) show the presence of amorphous halo in the range 20–30° 2θ, with an increase of Cu(NO_3_)_2_∙3H_2_O in the electrolyte. In addition, the anatase (TiO_2_) in crystal form for TiCu300 was also found. The remaining signal in all samples corresponds to titanium (matrix).

**Figure 7:**
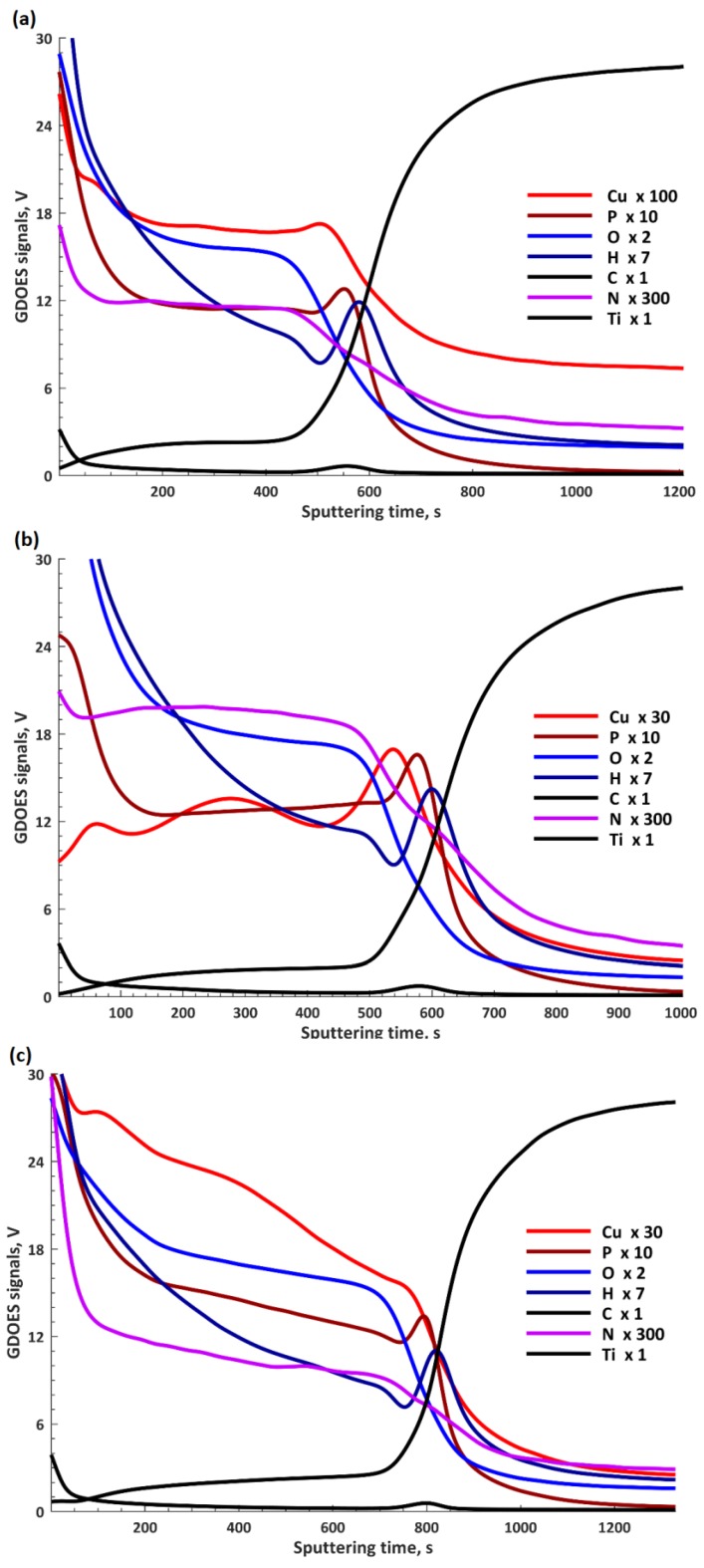
The GDOES depth profiles of the three coatings: TiCu300 (**a**), TiCu450 (**b**), and TiCu600 (**c**).

**Figure 8:**
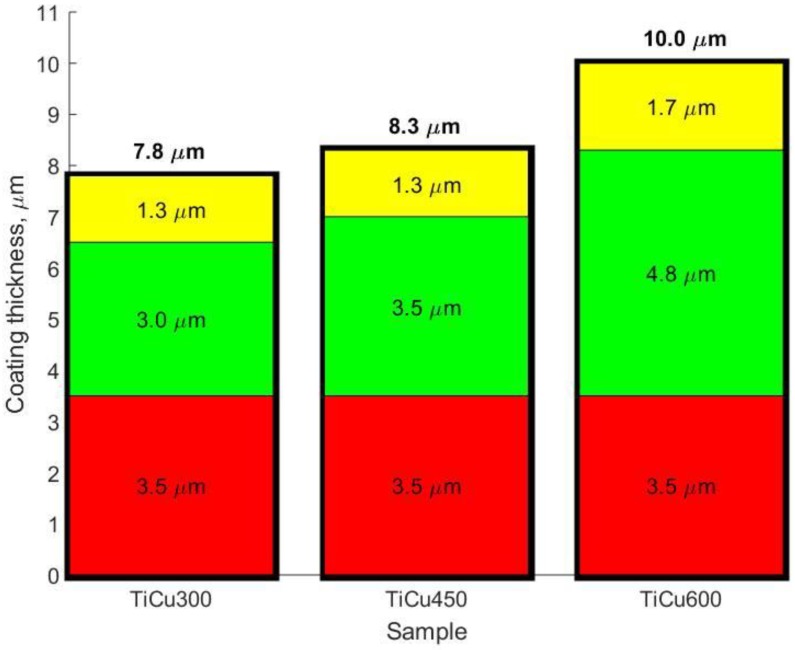
Layer thicknesses of the porous coatings determined on the basis of the GDOES analysis for the first stage of the studies; yellow color—porous layer, green color—semi-porous layer, and red color—transient layer.

In Figure 7, the results obtained by GDOES are presented and form a basis for which a three-layer model (Figure 8) can be used to describe these elemental profiles. The GDOES signals for copper, phosphorus, oxygen, hydrogen, carbon, and nitrogen follow a downward trend, whereas for titanium, a rising signal was registered. It should be noted that the GDOES signals for oxygen, hydrogen, carbon, and nitrogen registered in these layers ought to be treated as the compounds linked both to the coating and its organic contaminations, as also suggested by the XPS spectra obtained. The thicknesses of the top and porous layers changed from 1.3 μm (TiCu300) up to 1.7 μm (TiCu600), while the second semi-porous layers increased from about 3.0 μm (TiCu300) up to 4.8 μm (TiCu600). As can be observed, the third transition layers maintained approximately the same thickness for all samples, equal to 3.5 μm. The GDOES signals of copper, phosphorus, oxygen, hydrogen, carbon, and nitrogen in the first (top) layers of all studied samples are non-growing in character as a function of the GDOES etching time, whereas the titanium signal is a non-decreasing function during etching. The inner (second) layers, connecting the coating with titanium, have local maxima which may be observed in the spectra of copper, phosphorus, hydrogen, and carbon, probably connected with the formation of the compounds containing copper and hydroxyphosphate groups and organic contaminations. One should also admit that the GDOES signal for titanium in the range of the transition layer has a rising trend. To sum up, it can be concluded that the range of thicknesses of fabricated PEO coatings was from 7.8 μm (TiCu300) to 10.0 μm (TiCu600).

**Figure 9:**
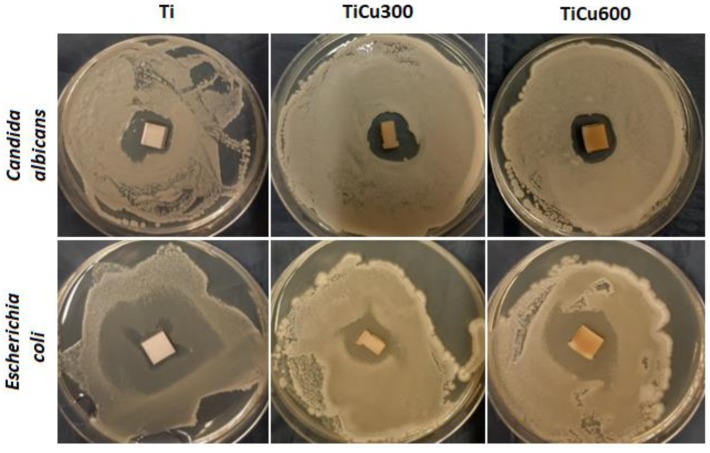
Sensitivity assessment of tested microorganisms applied on coatings.

In our study, both fungal and bacterial cells were observed to be sensitive to the coatings (Figure 9). Identified zones of inhibition of microbial growth around tested coatings were the assessment criteria. Antimicrobial activity of prepared samples results either from chemical compounds which are released into the around-implant environment, or from composites, which do not release the substance but act statically on microorganisms. In most cases, these compounds are characterized by having a positive charge on their surface, so they have a strong affinity to the negatively polarized cell wall of fungus and bacteria. After contact in each case, the growth of osmotic pressure in the cell and a change in the permeability of its wall lead, consequently, to lysis of the microorganism [17].

**Figure 10:**
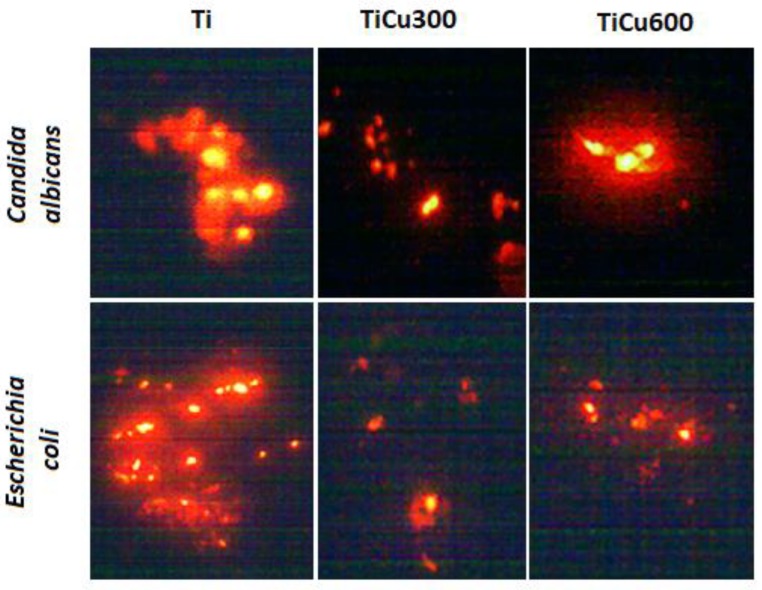
Example fields of adhered cells to reference sample (titanium) and fabricated coatings.

**Figure 11:**
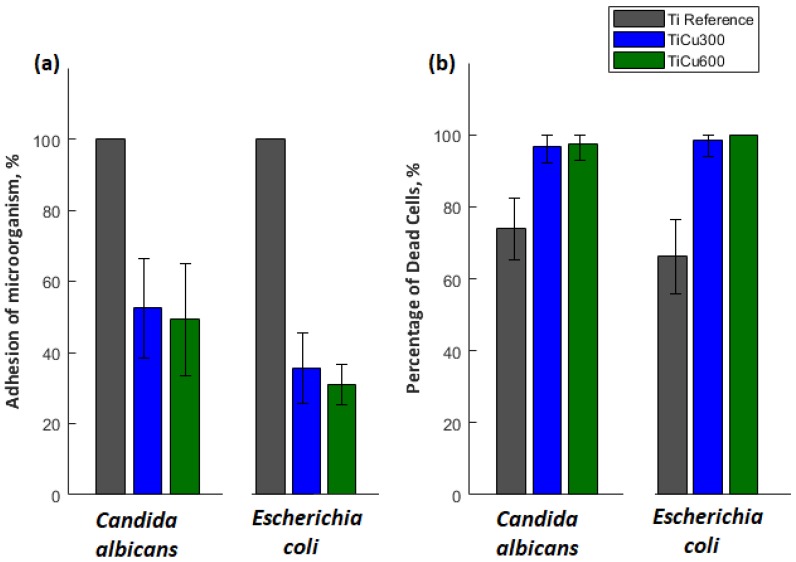
Quantitative analysis of microorganism adhesion to the coatings (**a**) and percentage of dead cells (**b**).

The obtained results indicate that all tested microorganisms are less likely to adhere to the tested coatings compared to the reference sample, which was the titanium. The smallest number of microorganisms was found on the coating TiCu600, while the highest number of microorganisms adhering to the tested samples was observed on the titanium (Figure 10), shown quantitatively in Figure 11a. It was also discovered that the samples enriched in copper had high antibacterial and fungicidal activity in relation to the reference (pure titanium) samples, presented in Figure 11b. It was observed that there were far less live bacteria than dead bacteria, which is extremely important for possible use of these materials in implantology. The impact of substrate material, surface roughness, and chemical composition on microbial adhesion are not exactly due to universal principles and differ between microorganism species [18].

## 4. Discussion

In this work, we showed that porous coatings may be fabricated in solutions based on 85 wt% orthophosphoric acid and copper(II) nitrate(V) trihydrate. Our experiments showed that it is possible to fabricate a porous coating using 300–600 g Cu(NO_3_)_2_∙3H_2_O in 1 dm^3^ H_3_PO_4_. The 3D roughness parameters were selected in consideration of what might be valid for porosity determination of the outer PEO coating, such as *Sa* and *Sp*, which were in the range 1.17–1.90 μm and 7.62–13.91 μm, respectively. The thicknesses of fabricated coatings changed from 7.8 up to 10.0 μm, which should be enough in case of biomedical application. It was also found out that all obtained coatings may be subdivided into three sublayers: the top sublayers (1.3–1.7 μm), which are porous, the semi-porous sublayers (3.0–4.8 μm), and the transition sublayers (3.5 μm).

The EDS and XPS studies have shown that the chemical composition in the whole volume of PEO coatings is not uniform. This can be concluded based on Cu/P ratios which are different for the whole volume of the coatings (0.05–0.12) and their top nano-layers (0.17–0.24). Such results suggest that the outer and porous part of coatings contain more copper compounds, which are mostly amorphous. In addition, it was recorded that the top nano-layer contains mostly titanium (Ti^4+^) and copper (Cu^+^, Cu^2+^) phosphates and/or diphosphates.

The most important fact is that the top nano-layer of coating is enriched more with the expected elements (approximately double for the TiCu600 sample), which results in its antibacterial and fungicidal effect. However, the biological studies proved that there are not any significant differences in antibacterial and fungicidal properties between TiCu300 (0.54 at% Cu) and TiCu600 (0.72 at% Cu). Additionally, it was found that the adhesions of fungi (*C. albicans*) and bacteria (*E. coli*) to substrate were significantly smaller than that observed for titanium reference. Based on this information, it can be concluded that for practical use the amount of copper(II) nitrate(V) trihydrate in electrolyte can be limited to 300 g per 1 dm^3^. It is generally known that copper has, in addition to bactericidal and fungicidal effects, a negative effect, e.g., on osteoblasts or other tissues. Accordingly, a smaller amount of that element inside the coatings should be desired; however, copper ions should be distributed in the entire volume of the fabricated coatings.

## 5. Conclusions

The following conclusions may be formulated:

- It is possible to fabricate porous coatings enriched in copper with the use of an average voltage equaling 450 V_RMS_ (ripple voltage 92 Vpp, 300 Hz), under a PEO process, in electrolytes based on concentrated orthophosphoric acid with the addition of copper(II) nitrate(V) trihydrate.
- In the obtained PEO coatings, one may distinguish three layers: porous (i), semi-porous (ii), and transient (iii); the porous layers are about two times thinner than the semi-porous and the transient layers.
- The formation of porosity may be described by two processes connected with the formulation of the primary porosity and, on that basis, of the secondary, which has more developed surface stereometry.
- The higher concentration of Cu(NO_3_) _2_∙3H_2_O in the electrolyte, the higher the roughness of the coatings.
- The 3D roughness parameters (Sa/Sp) of obtained samples fabricated in the electrolyte with 300–600 g/dm3 Cu(NO3)2 were in the range 1.17 μm/7.62 μm to 1.90 μm/13.91 μm.
- The thicknesses of PEO coatings obtained in the electrolyte with 300–600 g/dm3 Cu(NO3)2 were in the range 7.8 μm up to 10 μm.
- The higher the concentration of Cu(NO3) 2∙3H2O in the electrolyte, the higher the Cu/P ratio.
- The surface of coating with the top Cu/P ratio (10 nm) contains more copper (0.17–0.24) than the whole coating (0.05–0.12).
- The top nanolayer of coating contains phosphates and/or diphosphates as well as titanium (Ti^4+^) and copper (Cu^+^ and/or Cu^2+^).
- The atomic concentration of copper in the range 0.54 to 0.72 at% in the top surface of PEO coatings resulted in antibacterial and fungicidal properties; the higher amount of Cu^+^/Cu^2+^ in coatings, the better the antibacterial and fungicidal effect.
- The obtained phosphate-based PEO coatings with copper distributed in its volume should be qualified as antibacterial and antifungal, with application as transition layers between implant and tissues.

## Figures and Tables

**Table 1:** Abbreviated sample names

Abbreviation	Electrolyte Composition
TiCu300	1dm^3^ H_3_PO_4_ with 300g Cu(NO_3_)_2_∙3H_2_O
TiCu450	1dm^3^ H_3_PO_4_ with 450g Cu(NO_3_)_2_∙3H_2_O
TiCu600	1dm^3^ H_3_PO_4_ with 600g Cu(NO_3_)_2_∙3H_2_O

H_3_PO_4_ obtained from P.P.H. STANLAB Sp.J., Lublin, Poland. Cu(NO_3_)_2_∙3H_2_O obtained from CHEMPUR, Piekary Śląskie, Poland.

## References

[1] Alsaran A., Purcek G., Hacisalihoglu I., Vangolu Y., Bayrak Ö., Karaman I., Celik A. (2011). Hydroxyapatite production on ultrafine-grained pure titanium by micro-arc oxidation and hydrothermal treatment. Surf. Coat. Technol..

[2] Wan H.-Y., Zhu R.-F., Lu Y.-P., Xiao G.-Y., Ma J., Yuan Y.F. (2013). Preparation and mechanism of controllable micropores on bioceramic TiO_2_ coatings by plasma electrolytic oxidation. Surf. Rev. Lett..

[3] Wang Y.M., Guo J.W., Zhuang J.P., Jing Y.B., Shao Z.K., Jin M.S., Zhang J., Wei D.Q., Zhoua Y. (2014). Development and characterization of MAO bioactive ceramic coating grown on micro-patterned Ti6Al4V alloy surface. Appl. Surf. Sci..

[4] Wang X., Qu Z., Li J., Zhang E. (2017). Comparison study on the solution-based surface biomodification of titanium: Surface characteristics and cell biocompatibility. Surf. Coat. Technol..

[5] Zhu W., Fang Y.-J., Zheng H., Tan G., Cheng H., Ning C. (2013). Effect of applied voltage on phase components of composite coatings prepared by micro-arc oxidation. Thin Solid Films.

[6] Zhao D., Lu Y., Wang Z., Zeng X., Liu S., Wang T. (2016). Antifouling properties of micro arc oxidation coatings containing Cu_2_O/ZnO nanoparticles on Ti6Al4V. Int. J. Refract. Metals Hard Mater..

[7] Yao X., Zhang X., Wu H., Tian L., Ma Y., Tang B. (2014). Microstructure and antibacterial properties of Cu-doped TiO_2_ coating on titanium by micro-arc oxidation. Appl. Surf. Sci..

[8] Rokosz K., Hryniewicz T., Matysek D., Dudek L., Malorny W. (2015). SEM and EDS analysis of nitinol surfaces treated by plasma electrolytic oxidation. Adv. Mater. Sci..

[9] Rokosz K., Hryniewicz T., Raaen S. (2016). Development of plasma electrolytic oxidation for improved Ti6Al4V biomaterial surface properties. Int. J. Adv. Manuf. Technol..

[10] Rokosz K., Hryniewicz T., Dudek L., Matysek D., Valicek J., Harnicarova M. (2016). SEM and EDS Analysis of Surface Layer Formed on Titanium after Plasma Electrolytic Oxidation in H_3_PO_4_ with the Addition of Cu(NO_3_)_2_. J. Nanosci. Nanotechnol..

[11] Rokosz K., Hryniewicz T., Kacalak W., Tandecka K., Raaen S., Gaiaschi S., Chapon P., Malorny W., Matysek D., Dudek L. (2018). Characterization of Porous Phosphate Coatings Enriched with Calcium, Magnesium, Zinc and Copper Created on CP Titanium Grade 2 by Plasma Electrolytic Oxidation. Metals.

[12] Rokosz K., Hryniewicz T., Raaen S., Matysek D., Dudek L., Pietrzak K. (2018). SEM, EDS, and XPS characterization of coatings obtained on titanium during AC plasma electrolytic process enriched in magnesium. Adv. Mater. Sci..

[13] Rokosz K., Hryniewicz T., Gaiaschi S., Chapon P., Raaen S., Matysek D., Dudek L., Pietrzak K., Malorny W. (2019). Fabrication and Characterisation of Porous Coatings Enriched with Copper on CP Titanium Grade 2 under Plasma Electrolytic Oxidation. Teh. Vjesn. Tech. Gaz..

[14] Rokosz K., Hryniewicz T., Gaiaschi S., Chapon P., Raaen S., Matysek D., Dudek L., Pietrzak K. (2018). Novel Porous Phosphorus-Calcium-Magnesium Coatings on Titanium with Copper or Zinc Obtained by DC Plasma Electrolytic Oxidation: Fabrication and Characterization. Materials.

[15] Rokosz K., Hryniewicz T., Raaen S., Chapon P., Dudek L. (2017). GDOES, XPS, and SEM with EDS analysis of porous coatings obtained on titanium after plasma electrolytic oxidation. Surf. Interface Anal..

[16] Rokosz K., Hryniewicz T., Raaen S., Chapon P. (2016). Investigation of porous coatings obtained on Ti-Nb-Zr-Sn alloy biomaterial by plasma electrolytic oxidation: Characterisation and modelling. Int. J. Adv. Manuf. Technol..

[17] Półtorak K., Podlewska M., Szram A., Sokołowski J., Łukomska–Szymańska M. (2016). Composite materials with antimicrobial properties—Literature review. Stomatol. Prakt. Pol. Engl. J. Dent..

[18] Wassmann T., Kreis S., Behr M., Buergers R. (2017). The influence of surface texture and wettability on initial bacterial adhesion on titanium and zirconium oxide dental implants. Int. J. Implant Dent..

